# Exclusion of latecomers yields a patchwork of viral subpopulations within hosts

**DOI:** 10.1371/journal.pbio.3001994

**Published:** 2023-02-09

**Authors:** Anice C. Lowen, Lucas M. Ferreri

**Affiliations:** Department of Microbiology and Immunology, Emory University School of Medicine, Atlanta, Georgia, United States of America

## Abstract

At the level of individual cells, infection by late-arriving viruses is blocked. At the level of tissues, genetically distinct patches of infection form. Is there a connection? This Primer explores a new PLOS Biology study suggesting that the answer is "yes."

## Teaser

Viruses arriving late to an individual cell are blocked from replicating, an effect called superinfection exclusion. A study in *PLOS Biology* indicates that this exclusion at the level of individual cells gives rise to a heterogenous landscape of infection within a host.

Superinfection exclusion is a phenomenon in which a virus replicating within a cell blocks secondary infection of that cell by viruses of the same species [1]. Recently, in *PLOS Biology*, Sims and colleagues uncovered the role of superinfection exclusion in producing spatially discrete viral subpopulations [2]. While exclusion has long been recognized, its implications for the spatial structure of viral populations during in vivo infections had not been explored. This work offers timely mechanistic insight into the forces shaping the genetic diversity and evolution of viral populations within hosts.

**Fig 1:**
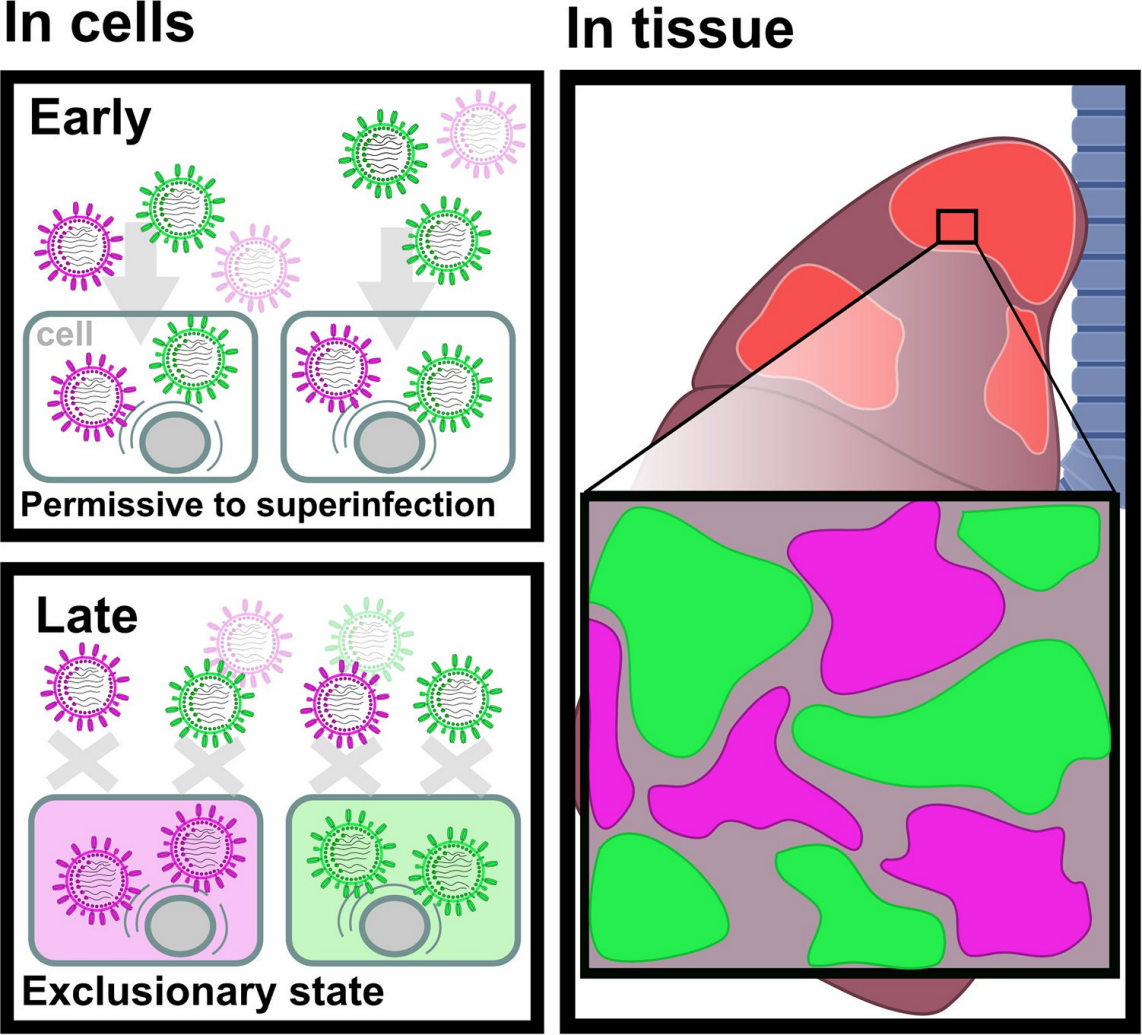
Superinfection exclusion within cells leads to spatial patterning within tissues. At early stages of the viral cycle, cells are permissive to infection of more than one virus, whereas in later stages, superinfection exclusion is set, impeding the reception of other viruses by the cell. Recent work in *PLOS Biology* shows that superinfection exclusion leads to a mosaic of discrete virus populations at the tissue level.

Superinfection exclusion is a temporal effect: Once a cell is infected, a clock starts ticking. The cell remains open to secondary infection for a period of hours but then adopts an exclusionary state in which newly arriving viruses are not able to initiate sustained replication [3,4]. Sims and colleagues [2] show that this temporal effect gives rise to strong spatial patterning (Fig 1). Their data support a model in which only viruses that are nearby in space—because they are progeny of the same infected cell, for example—can readily coinfect within the time window that precedes exclusion. At later times of infection, expanding viral populations may converge; however, at this stage, most cells in an expanding focus would already be in an exclusionary state, such that coinfection cannot extend beyond the leading edge. The result is sectoring of the infected tissue into foci, each of which comprises closely related viruses.

This model offers mechanistic insight relevant to recent reports that describe spatially discrete subpopulations within hosts infected with genetically diverse influenza A viruses. One such report monitored the dynamics of a barcoded influenza A virus library [5]. The second tracked genetic diversity generated de novo through reassortment [6]. Both saw that distinct sites within the respiratory tract harbored subpopulations that were genetically dissimilar. In the case of the barcoded virus, thousands of variants were instilled into the nose of ferrets and many genotypes subsequently dispersed to the lung; however, only a small number became established in each lung lobe. Owing to this tight genetic bottleneck, the genotypes predominating at each site differed. Similarly, in swine, viral variants generated through reassortment formed spatially distinct subpopulations, typically with only a few novel genotypes detected at each site. In light of the findings of Sims and colleagues [2], both the low diversity within each site and the observed lack of mixing between them can be explained by superinfection exclusion: Only the first viruses to become established at a site are efficiently propagated, with any subsequently arriving viruses unable to penetrate.

Superinfection exclusion is thought to have evolved across many virus families because it can allow an individual virus to propagate its genome in the absence of competition. However, excluding coinfection would eliminate the beneficial interactions that result from delivery of multiple viral genomes to the same cell [7,8]. For influenza A viruses, these interactions include functions as fundamental as the reconstitution of a complete viral genome from parental viruses with incomplete genomes [7,9]. For this reason, it is likely critical for viral propagation that the block to superinfection occurs only after a matter of hours, allowing time for nearby viruses to infect the same cell.

Another important implication of superinfection exclusion is its potential to limit viral genetic exchange. For influenza A viruses, such exchange happens readily within coinfected cells through the reassortment of gene segments. While reassortment likely occurs in all coinfected cells, it will only generate diversity when distinct parental genomes are involved. Thus, consequential reassortment is reliant on viruses mixing within infected hosts. The observed spatial heterogeneity of influenza A virus populations indicates that such mixing is constrained [2,5,6]. By reducing opportunity for the generation of genetic diversity, this constraint diminishes adaptive potential.

Other consequences of superinfection exclusion for viral evolution are likely to occur but are more challenging to anticipate. Although as-yet unexplored, it may be the case that fitter viruses trigger superinfection exclusion more rapidly. If so, shielding a cell from further incoming viruses would help to limit the propagation of less fit variants, thus helping to maintain population fitness. This model assumes that multiple variants colonize an area of tissue together, allowing competition among them to ensue. Indeed, tracking of barcoded influenza A viruses offers support for this idea [5]. Conversely, where long-distance dispersal is rare, superinfection exclusion would be expected to promote founder effects. Here, certain genotypes would dominate simply because they were at the right place at the right time. Such chance events would act to weaken selection, for example, by allowing the propagation of mildly deleterious variants. According to this model, superinfection exclusion may represent a short-sighted evolutionary strategy, with benefits to individual viruses coming at a cost for viral populations [10]. Of note, these concepts are consistent with viral evolutionary dynamics observed within influenza virus-infected individuals: Viral diversity within a host is typically low and stochastic effects appear dominant over selection [11]. Superinfection exclusion may be one driver of these effects.

Although the work of Sims and colleagues [2] offers a major advance by linking spatial structure within hosts to superinfection exclusion, many questions persist. While their study and related ones focused on the lower respiratory tract, the upper respiratory tract is an important site for influenza A virus replication and onward transmission. Thus, it will be relevant to determine how the extent of viral mixing varies between sites of differing anatomical architecture and cell type composition. Each of the studies discussed herein furthermore monitored the dynamics of genetically distinct viruses of comparable fitness. The implications for selection of superinfection exclusion and the spatial heterogeneity that it promotes therefore remain untested. Related to this point, the extent to which the timing and potency of superinfection exclusion vary across influenza A virus strains is unclear, as is the relationship of any such phenotypic variation to viral fitness. Are more fit viruses better at establishing superinfection exclusion? Will a virus that more rapidly establishes superinfection exclusion have a competitive advantage? Answers to these questions are within reach, thanks to the tools of modern molecular virology, and will bring critical new insights into the evolutionary dynamics of viral populations within hosts.
